# Amyl Nitrite

**Published:** 1894-08

**Authors:** 


AMYL NITRITE is a physiological antidote to cocaine, and should always be at hand when making applications of the latter.

